# Timely and atomic-resolved high-temperature mechanical investigation of ductile fracture and atomistic mechanisms of tungsten

**DOI:** 10.1038/s41467-021-22447-y

**Published:** 2021-04-13

**Authors:** Jianfei Zhang, Yurong Li, Xiaochen Li, Yadi Zhai, Qing Zhang, Dongfeng Ma, Shengcheng Mao, Qingsong Deng, Zhipeng Li, Xueqiao Li, Xiaodong Wang, Yinong Liu, Ze Zhang, Xiaodong Han

**Affiliations:** 1 Beijing Key Laboratory of Microstructure and Property of Advanced Materials, Beijing University of Technology, Beijing, China; 2 College of Materials Science and Engineering, Key Laboratory of Advanced Functional Materials, Education Ministry of China, Beijing University of Technology, Beijing, China; 3 Department of Fundamental Sciences, Chinese People’s Armed Police Force Academy, Langfang, China; 4 Department of Mechanical Engineering, The University of Western Australia, Perth, WA Australia; 5 State Key Laboratory of Silicon Materials and Department of Materials Science and Engineering, Zhejiang University, Hangzhou, China

**Keywords:** Materials science, Structural materials

## Abstract

Revealing the atomistic mechanisms for the high-temperature mechanical behavior of materials is important for optimizing their properties for service at high-temperatures and their thermomechanical processing. However, due to materials microstructure’s dynamic recovery and the absence of available in situ techniques, the high-temperature deformation behavior and atomistic mechanisms of materials are difficult to evaluate. Here, we report the development of a microelectromechanical systems-based thermomechanical testing apparatus that enables mechanical testing at temperatures reaching 1556 K inside a transmission electron microscope for in situ investigation with atomic-resolution. With this unique technique, we first uncovered that tungsten fractures at 973 K in a ductile manner via a strain-induced multi-step body-centered cubic (BCC)-to-face-centered cubic (FCC) transformation and dislocation activities within the strain-induced FCC phase. Both events reduce the stress concentration at the crack tip and retard crack propagation. Our research provides an approach for timely and atomic-resolved high-temperature mechanical investigation of materials at high-temperatures.

## Abstract (web summary)

High-temperature deformation of materials is challenging to evaluate. Here the authors develop a novel device that allows atomic resolved in situ high temperature mechanical tests inside a transmission electron microscope and reveal ductile fracture of a single crystal tungsten deformed at 973 K.

## Introduction

The mechanical properties and deformation behavior of structural materials at high-temperatures are fundamental and critically important for a wide range of applications, such as turbine engines operating at high-temperatures and thermomechanical processing of metallic materials. Many refractory metals, such as tungsten and molybdenum, are important constituents of structural materials. They are brittle at ambient temperatures and may exhibit ductility only at very high-temperatures. However, studying their ductile behavior and particularly understanding the atomic mechanisms of these behavior are extremely challenging. This has resulted in a glaring knowledge gap in our understanding of these metals and hampered our ability to process and use them in applications. Recently, the development of in situ transmission electron microscopy (TEM) has played an important role in revealing the deformation and fracture mechanisms of materials^1–3^. Based on the high-resolution and real-time observation characteristics of this technique, novel deformation and fracture behavior have been successfully revealed, including dislocation-dominated deformation^4^, twinning-dominated deformation^5^, mechanical annealing^1^, phase transformation^6^ and size effects^7^ at room temperature. However, the development of in situ TEM technique at high-temperatures, particularly for visualization at the atomic-scale, has been prevented due to technical limitations (including the extremely small chamber in transmission electron microscopes, the precise control/measurement of temperature, the precise loading of forces and displacements at high-temperatures^8^). Many researches have been carried out on improving the temperature and resolution of in situ mechanical tests. Atomic-resolved in situ experimental device that can achieve mechanical testing^9,10^ when the temperature approaches 600 K was developed. Some devices allow the stress and strain of the sample to be measured in situ during TEM examination^8,10^. Such temperature range, however, is not sufficient to cause new events or deformation mechanisms for many metals, particularly refractory metals like W. To date, atomic-resolved in situ mechanical testing inside high-resolution TEM above 600 K remains a challenge. Thus, the research on the deformation and atomic-scale fracture mechanisms of materials at high temperatures is nearly still in the theoretical category. Therefore, it is natural to ask whether these deformation mechanisms of the materials at high temperatures under real experimental conditions are different from those in previous theoretical studies.

Tungsten (W) plays an important role in high-temperature applications. It is a special metal due to its unique properties, including the highest melting temperature, the lowest thermal expansion coefficient, the highest Young’s modulus, and the highest strength at above 1600 °C among all metals. The high melting point and excellent high-temperature mechanical strength render W a candidate material for many high-temperature applications^11,12^. However, W is highly brittle at room temperature^13^ with a high brittle-ductile transition (BDT) temperature of 300 °C^13–15^. This makes it very difficult to study the mechanisms of ductility in W, particularly at the atomic-scale due to the experimental challenges in achieving such conditions in a microscope^13,15–18^.

Whether a metal fractures in a brittle or ductile manner is determined by the relative ease of cleavage and dislocation movement. The ease of dislocation movement is described by the Peierls stress (*σ*_p_), which is determined by the dislocation core width (*ξ*) (the minimum distance between two parallel dislocations)^19,20^. Rice and Thomson^21^ conducted a survey of many body-centered cubic (BCC) and face-centered cubic (FCC) metals for the correlation between the *ξ* values and the fracture behavior and proposed an empirical criterion to predict the fracture mode as *ξ* > *ξ*_c_ for ductile fracture, and vice versa. The value of *ξ*_c_ also represents the minimum distance for a dislocation to remain stable from a nearby crack tip, or below which the dislocation becomes unstable and becomes attracted to the crack tip. Their survey indicates that for most FCC metals *ξ*_FCC_ > *ξ*_c_,_,_ whereas for most BCC metals *ξ*_BCC_ < *ξ*_c_ under ambient conditions. The magnitude of the critical distance *ξ*_c_ for a dislocation to remain stable within the vicinity of a crack tip is dependent on the temperature^22^. Generally, *ξ*_c_ decreases with decreasing temperature^23,24^. This provides an opportunity for *ξ*_BCC_ > *ξ*_c_, i.e., for BCC metals to fracture in a ductile manner. This is the reason for the BDT of many BCC metals. Ductile fracture of W can be achieved by increasing the temperature to above 743 K^13,25,26^. For some BCC metals, such as Nb^27^ and Mo^6,28^, the deformation-induced BCC→FCC phase transformation at the crack tip provides another mechanism to induce ductile fracture. The induced FCC phase is intrinsically more ductile than the original BCC structure, thus decreasing the tendency for brittle fracture. These transformations are generally not predicted or permitted in equilibrium thermodynamics for stress-free conditions^29–31^, but can be induced by deformation in the specific environment at the crack tips in these metals^6^.

Crack tip induced phase transformations have been experimentally observed in BCC structural metals^6,27,28,32^, such as Nb, Mo, Ta and ceramics^33,34^, by means of TEM during in situ deformation. However, no such BCC→FCC transformation has been observed in W. Molecular dynamics simulations on single-crystal W have revealed that such a transformation is possible above 673 K^35,36^. To verify this prediction, several TEM and scanning electron microscopy (SEM) studies have been conducted, but none have revealed this transformation for W^5,13,16,18,37^. One possible explanation is that the FCC structure induced mechanically at the crack tip is thermodynamically unstable and readily reverts to the stable BCC phase once the mechanical load is removed^6^, thus making any ex situ techniques incapable of capturing the evidence. In addition, ex situ TEM sample preparation processes, whether by mechanical grinding and electrochemical polishing^38^ or focused ion beam milling^39^, inevitably causes structural disturbances and damage, which may easily trigger the reversion of the induced thermodynamically unstable FCC phase. Furthermore, the “transformation zone” at the crack tip is usually very small, typically ~10 nm, as in the cases of Si, Ge, SiC, and Al_2_O_3_^40^. All these results imply that in situ high-resolution transmission electron microscopy (HRTEM) examination with real-time mechanical testing at elevated temperatures is essential for capturing crack tip-induced phase transformations in W. This is obviously a large experimental challenge.

In this study, we develop an advanced technique with a custom-designed device that allows in situ deformation at up to 1556 K inside a transmission electron microscope with atomic-scale resolution. Using this unique technique, we reveal for the first time direct evidence of a strain-induced BCC→FCC phase transformation at crack tips and plastic activities within the FCC structure that was formed in W. The strain-induced phase transformation and the dislocation activities provide a two-fold deformation mechanism to resist brittle fracture and to improve the ductility of W.

## Results and discussions

### In situ atomic-resolution high-temperature mechanical testing system

**Fig. 1:**
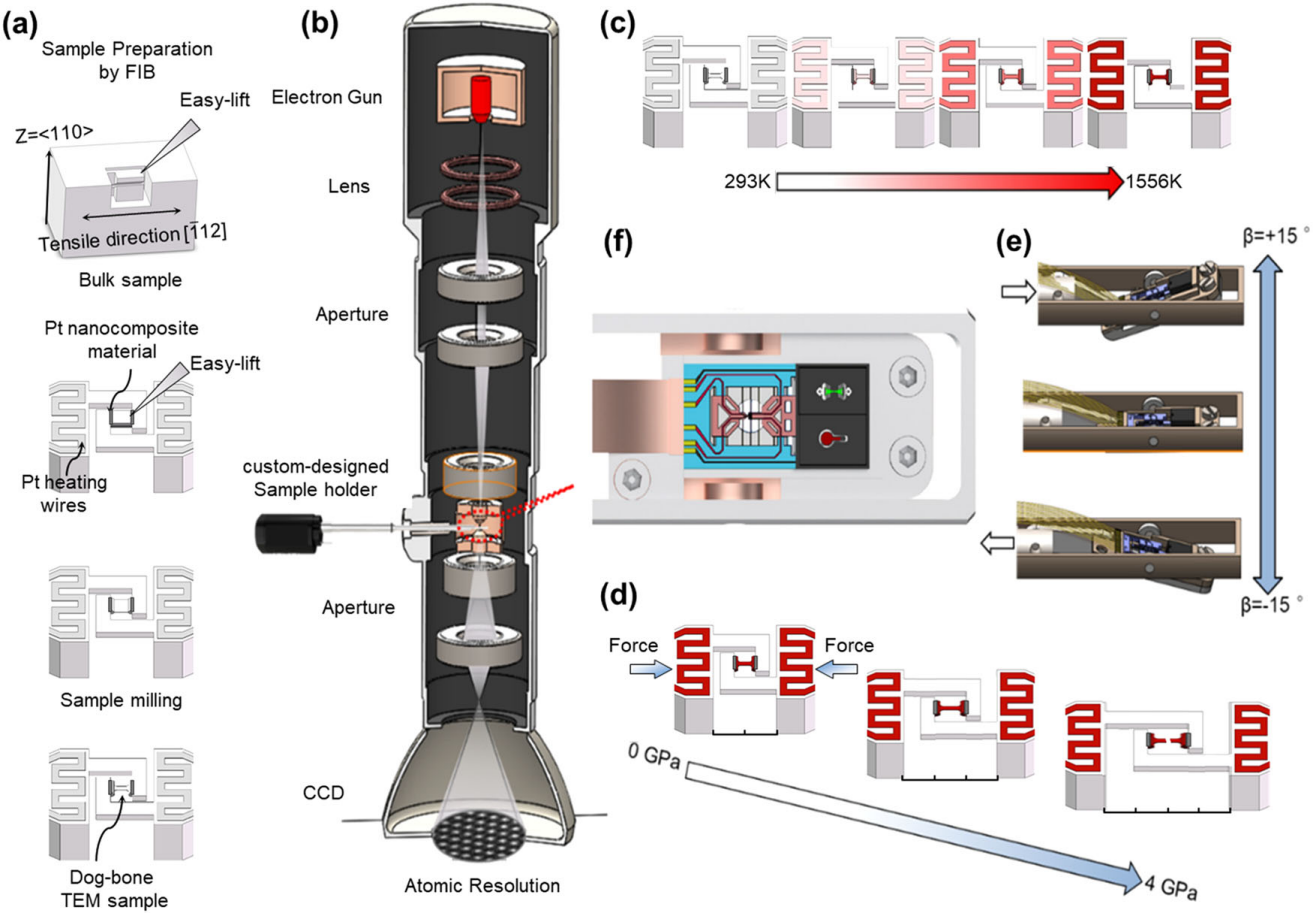
Atomic-resolved high-temperature mechanical testing system. Schematic of **a** The sample preparation and mounting procedure. **b** The specially designed thermomechanical test device inside the column of a high-resolution transmission electron microscope for in situ analysis. **c** The temperature control component. **d** The in situ deformation component. **e** The custom-designed double-tilt sample holder containing the temperature control and the in situ deformation components. **f** The details of the head of the sample holder.

In situ TEM observation of the fracture behavior of single-crystal tungsten at an elevated temperature was conducted using a custom-designed TEM sample holder device^41,42^, as shown in Supplementary Fig. 1a and Supplementary note 1. The device was able to heat the sample inside the microscope to a set temperature and deform it in tension, compression, bending or indentation or hold the load to induce creep while allowing the sample to be observed for microstructural changes at the atomic-scale in TEM mode. Figure 1 shows the design of the device and the TEM sample preparation and mounting procedure. The device contained two essential components, one for temperature control (component (c)) and one for applying deformation (component (d)). Heating of the sample was achieved by a MEMS Pt heating circuit constructed at both ends of the sample, as seen in Supplementary Fig. 1e. In the meantime, the Pt heating circuit also provides current and voltage feedback to track the supply power and to serve as the temperature monitor. The wide frontages of the Pt heating circuits help to assure a uniform and stable temperature field around the sample located in between them. Before the growth of the Pt heating circuit, an oxidized Cr adhesion layer (30 nm) was deposited on the Si substrate covered by a SiO_2_ dielectric barrier layer (300 nm). The oxidized Cr adhesion layer enhances the bonding strength between the substrate and Pt heating circuit. Therefore, the delamination of Pt heating wires can be effectively avoided at temperatures up to 1556 K. The oxidized Cr adhesion layers enable the MEMS heating chips with long stable servicing lives at different temperatures, e.g., at least 80 h at 1073 K. Temperature control during in situ deformation and TEM examination was achieved by controlling the electrical power supplied to the Pt heating wires based on a temperature-power function, which was empirically established and calibrated. The accuracy of temperature loading is up to ±0.1 K through voltage control. The electrical power supply to the heating circuits was controlled by the standard four-electrode method, as shown in Supplementary Fig. 2c and Supplementary note 1. The calibrations were conducted in vacuum chambers (better than 10^−4^ Pa) to mimic the environment inside a microscope, as seen in Supplementary Fig. 2a. The temperature calibrations were conducted through two steps: 1) By means of Raman Spectroscopy, the measurement was carried out at the tips of the single-crystal Si mounting arms for the sample, as indicated by the red dots seen in Supplementary Fig. 2c. Supplementary Fig. 2b shows the Raman stokes peak of Si measured at different supply powers to the heating elements. The peak positions are correlated to temperatures based on the peak position-temperature correlation of Si^43^. Then the heating power vs temperature calibration function of the device was empirically established, as shown in Supplementary Fig. 2d and Supplementary note 2. 2) The second step is to calibrate this function by directly observing the melting of pure Al and Cu samples induced by the device heating inside the TEM and SEM, respectively. For each kind of metal, multiple samples were used. The relative variations of melting temperature measurement among the multiple samples were about ±8 K for the pure Al samples and about ±9 K for the pure Cu samples. Therefore, the temperature measurement error bar is about ±9 K for the current research, as seen in Supplementary Fig. 2d and Supplementary note 2. Component (d) was a miniature device for applying force and displacement to the sample and was driven by a lead zirconate titanate (PZT) actuator. The PZT actuator was able to apply load up to 1 N with a resolution of 5 nN, corresponding to a stress of ~4 GPa for the sample size used and to produce a displacement up to 4 µm at a step size of 0.1 nm, corresponding to a strain resolution of ~1 × 10^−4^ relative to the sample size used. These two components were built on a miniature stage to form a thermomechanical integrated stage (TMIS)^42^, as shown in Supplementary Fig. 1c. The TMIS was fixed on a double-tilt sample holder (Fig. 1e), also developed in-house, as shown in Supplementary Fig. 1b. The holder allowed the sample to tilt about the longitudinal and transverse axes for ±15°. This capability is essential for good atomic-scale resolution, which requires precise alignment of the electron beam to a low-index zone axis of the sample during thermomechanical testing.

Sample fabrication was conducted by means of the focused ion beam (FIB) milling method using an FEI Helios 600i FIB instrument. Supplementary Fig. 3 and Supplementary note 3 show the process of sample preparation by FIB instrument. A [110] normal orientation miniature tensile sample was cut out of a tungsten single crystal into a typical dog-bone shape by easy lift in the FIB instrument (Fig. 1a). The sample was welded onto the in situ thermomechanical device by ion beam-induced deposition using a Pt nanocomposite material (nanometer-sized Pt metal crystals in an a-C matrix) inside the FIB instrument. After the FIB preparation process, the sample was cleaned again in a Nano Mill (Fischione Model 1040) using a low-energy Ar ion beam of 800 eV to remove the surface damage layer caused by Ga ions in the FIB process.

### In situ observation of the fracture propagation in W at 973 K

**Fig. 2:**
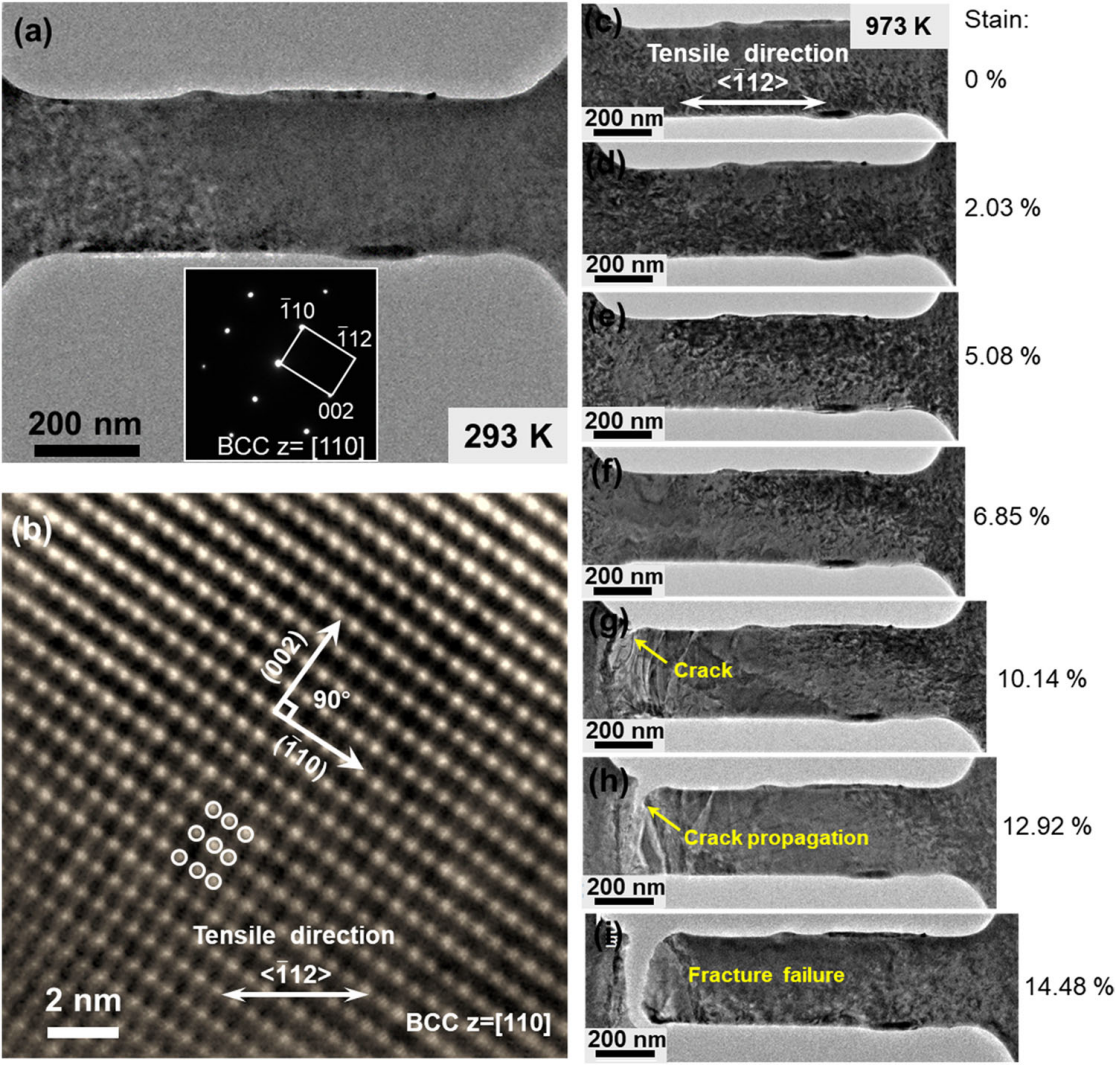
In situ TEM observation of tensile fracture of W at 973 K. **a** A TEM image of a dog-bone shaped single-crystal W specimen fabricated by FIB. The inset is a selected area electron diffraction pattern of the specimen, revealing the [110] plane normal and the near [$\overline 1$12] length directions. **b** High-resolution TEM image of the specimen at zero load. **c–i** Sequential TEM images of the specimen at different stages of the tensile deformation at 973 K.

Figure 2 presents an in situ TEM observation of the tensile deformation of a W single-crystal sample. Figure 2a is an TEM image of the dog-bone shaped sample mounted on the thermomechanical testing device. The sample had gauge section dimensions of 1200 × 300 × 60 nm. The sample was first heated to 973 K at a heating rate of 20 K/min inside the microscope and held for 15 min to stabilize the system before stretching to fracture. Supplementary Fig. 4 and Supplementary note 4 show the ANSYS simulation of the influence of sample loading on the temperature distribution of the heating zone. The results show a localized and isolated heating on the sample. Figure 2a shows a sample mounted on the thermomechanical testing device. The inset shows a selected area electron diffraction (SAED) pattern of the single-crystal sample, revealing its [110] orientation in the foil normal direction. The length direction of the tensile sample was close to [$\overline 1$12] (within an ~5° deviation). Figure 2b shows a high-resolution image of a local area of the sample at zero load, confirming the single-crystal orientation. Figure 2c–i show images of the sample at different stages of tensile deformation. The strains shown for the images were measured between the two ends of the gauge length of the sample. The sample was observed to have experienced a deformation up to a 5.08% strain without any sign of necking or cracking (Fig. 2c–e). Supplementary Fig. 5 and Supplementary note 5 shows the dislocation activities in the specimen during deformation at 973 K prior to the initiation of the first crack. During deformation up to 5.48%, the dislocation density continued to increase, as seen in Supplementary Fig. 5b–e. At 6.58% of strain, the first crack initiated, and the local stresses are immediately relaxed and dislocation density within the vicinity of the crack reduced, as evident in Supplementary Fig. 5f. This demonstrates that local dislocation activities are the main mechanism of deformation before the formation of cracks. The crack propagated gradually through the cross-section of the sample upon further stretching to full fracture at a 14.48% global strain (Fig. 2i) (the 14.48% global strain was measured after closing the gap of the rupture).

### Atomic-scale in situ observation of the BCC→FCC phase transformation near the crack tip under tensile loading at 973 K

**Fig. 3:**
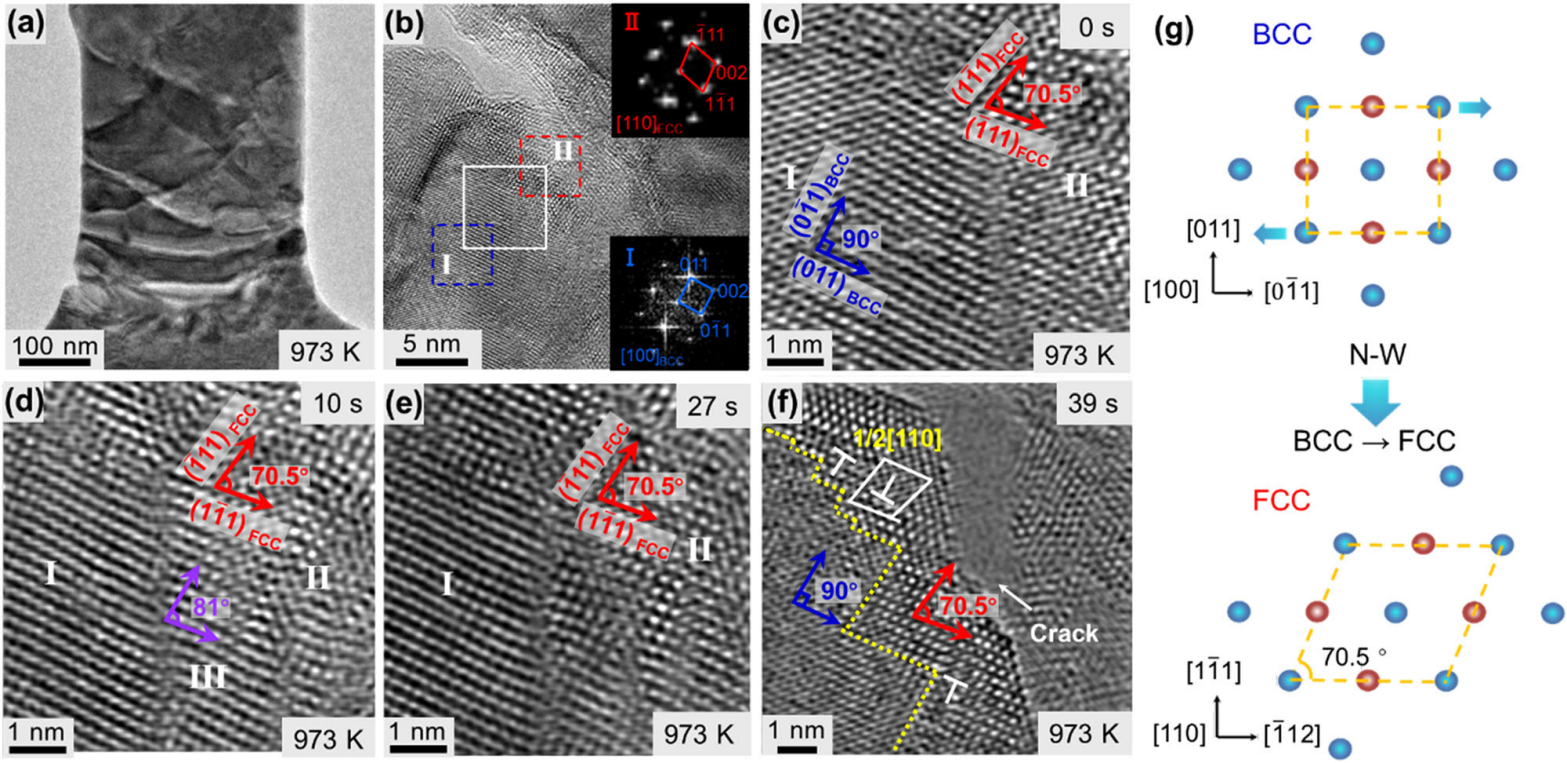
HRTEM observation of strain-induced BCC-FCC phase transformation. **a** A TEM bright-field image of the sample at the moment when a crack was initiated upon tensile deformation at 973 K. **b** A HRTEM image of the region around the crack tip. The insets are two Fast-Fourier Transform patterns of two areas (red dashed box and blue dashed box) in this region. **c**–**f** Time-resolved HRTEM images of the region within the white box in **b** showing BCC-FCC phase transformation at the crack tip at 973 K. Region I marked with the blue basic lattice vectors is [100]_BCC_ zone axis. Region II marked with the red basic lattice vectors is [110]_FCC_ zone axis. Region III marked with the purple basic lattice vectors is intermediate phase. **g** A crystallographic schematic of the BCC-FCC phase transformation by habit plane shear of the lattice.

Figure 3 shows detailed TEM images with atomic-resolution of the nucleation and propagation of the crack seen in Fig. 2. Figure 3a shows the sample with a newly formed crack (it is the same image shown in Fig. 2g). Figure 3b is a high-resolution image of the area around the crack. The insets are two fast-Fourier transform (FFT) patterns taken from the two regions inside the area. Pattern I is from Region I identified by the blue dashed box. It is indexed to the BCC structure with a [100]_BCC_ zone axis, apparently representing the metal in the new state near the crack tip. Pattern II is from Region II identified by the red dashed box, which is directly in front of the crack tip. The pattern is indexed to the FCC structure with a [110]_FCC_ zone axis, apparently representing a newly formed phase. Figure 3c shows the area identified by the white dashed box in (b) at an increased magnification. The area covers both the BCC region on the lower left and the FCC region on the upper right. The basic lattice vectors in Region I are 90° apart, conforming to the {110} relationship for the [100]_BCC_ zone axis, and those in Region II are 70.5° apart, conforming to the {111} relationship for the [110]_FCC_ zone axis. The (011)_BCC_ *d*-spacing in Region I was determined to be 2.250 Å. Compared to 2.215 Å for the stress-free state^44^, this corresponds to a local lattice strain of 1.58%. The ($\overline 1$11)_FCC_ *d*-spacing in Region II was determined to be 2.457 Å. Compared to 2.372 Å for the stress-free state^45^, this corresponds to a local lattice strain of 3.58%.

Figure 3d shows an image of the same area after 10 s of continued stretching at 0.1 nm/s from (c) (corresponding to approximately an increment of 0.08% global strain). A small portion of the [100] BCC structure in Region I transformed into a new structure (Region III). The two basic lattice vectors had *d*-spacings of 0.21 nm and 0.243 nm and are 81° apart. This structure cannot be indexed to any zone axis of either the BCC or FCC W and thus is regarded only as an intermediate state.

Figure 3e shows the area after another 17 s of stretching from (d) (corresponding to approximately an increment of 0.14% global strain). By this time, the intermediate phase in Region III evolved into a [110]_FCC_ structure and merged into Region II, completing the [100]_BCC_→[110]_FCC_ transformation. The crystallographic mechanism of the BCC→FCC transformation has been suggested to be lattice shear in [0$\overline 1$1]_BCC_ by 28.7% on (011)_BCC_ as the habit plane, as expressed in Fig. 3g^46^. This defined the lattice correspondence between the two phases as (100)_BCC_//(110)_FCC_ and (011)_BCC_// (1$\overline 1$1)_FCC_. This is consistent with the experimental evidence seen in Fig. 3c. This is known as the N-W model^46^. Figure 3f shows the area after another 12 seconds of stretching from (e) (corresponding to approximately an increment of 0.10% of global strain). By this time, the crack has propagated into the area, cutting into the FCC phase region. Dislocations can be clearly recognized within the FCC lattice.

During the above process, in addition to discrete changes to the lattice, such as the phase transformation and formation of dislocations, a continuous elastic lattice distortion was also observed. Local lattice distortions within the vicinity of the crack tip were characterized by means of the Peak Pairs Analysis (PPA)^47^ on the HRTEM images presented in Fig. 3c–e. The (010)_BCC_ *d*-spacing strains were determined relative to the value of 1.58 Å for the unstressed BCC W, and the results are presented in Supplementary Fig. 6a–c and Supplementary note 6. The dashed box in the strain map micrographs indicates an interface area between a [100]_BCC_ region and a [110]_FCC_ region. The average lattice strain of the [100]_BCC_ structure in the boxed area was found to increase during the continued deformation and reached a maximum of 3.8% before the occurrence of the BCC→FCC transformation. This strain corresponds to a lattice elastic stress of 14.57 GPa, based on the Young’s modulus of 380 GPa of W at 973 K^48^. Such a stress level far exceeds the usual bulk strength of W. This implies that the deformation-induced BCC→FCC phase transformation can only occur in the environment around a crack tip and not in the bulk of the matrix.

### Molecular dynamics (MD) simulation of the [110]_BCC_→[110]_FCC_ phase transformation

**Fig. 4:**
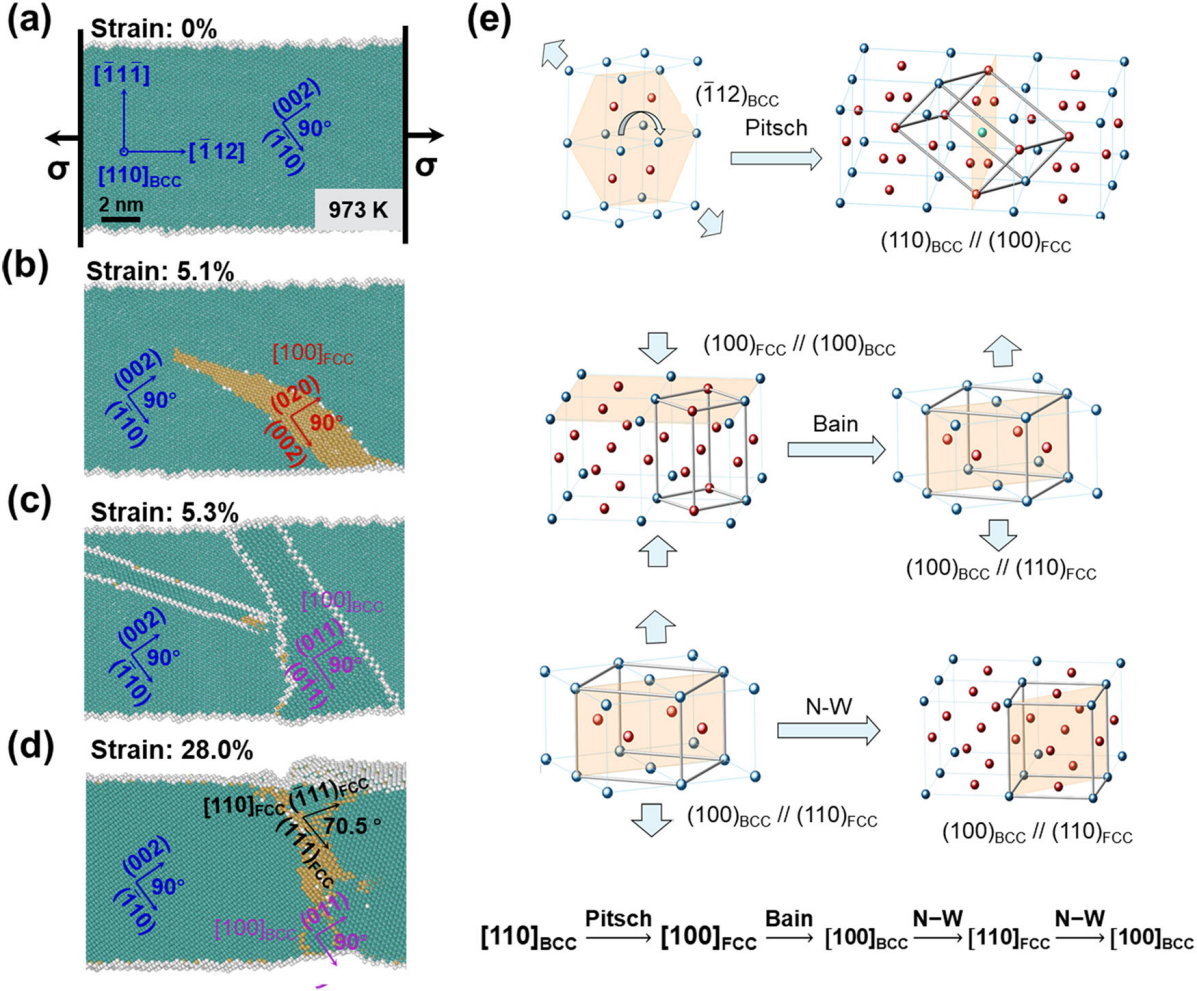
MD simulation of structural changes of W single crystal at 973 K. **a**–**d** Snapshots of the W structure in [110] viewing direction during the process of stretching, demonstrating the transformation process of [110]_BCC_-[100]_FCC_-[100]_BCC_-[110]_FCC_. **e** Crystallographic mechanisms of the pathway for the above transformation process. The atoms of the BCC structure are colored green and the atoms of FCC structure are colored yellow. The regions marked by different color basic lattice vectors show different lattice structures, blue: [110]_BCC_, red: [100]_FCC_, purple: [100]_BCC_, black: [110]_FCC_.

The evidence presented in Fig. 3 demonstrates that the [110]_BCC_→[100]_BCC_ transformation is not a simple step but a complex process involving intermediate steps. The actual process of the transformation cannot be captured experimentally because it is a diffusionless lattice distortion process^46^ that always propagates as mechanical waves at the speed of sound within the solid^49^. To understand the process, a MD simulation was carried out under the same experimental conditions, as presented in Fig. 4. Figure 4a shows a W supercell at 973 K. A supercell of 30 × 10 × 10 nm in the [$\overline 1$12], [$\overline 1$1$\overline 1$], and [110] (normal) orientation containing approximately 200,000 atoms was used for the simulation. After temperature equilibration at 973 K, tension along [$\overline 1$12]_BCC_ was applied at a constant strain rate up to a 30% global strain. Figure 4b shows the supercell after 510 ps of straining (corresponding to 5.1% of global strain). It is seen that part of the [110]_BCC_ structure transformed to the [100]_FCC_ structure. The habit plane was (002)_BCC_. This produced a lattice correspondence of (110)_BCC_//(100)_FCC_, which conforms to the model by Pitsch^50^. After 530 ps of straining (corresponding to 5.3% of global strain), the [100]_FCC_ structure converted to the [100]_BCC_ structure, as shown in Fig. 4c. This process conforms to the model by Bain^51^. The final step of the transformation from the [100]_BCC_ to [110]_FCC_ structure occurred after 2800 ps of straining (corresponding to 28.0% of global strain), as presented in Fig. 4d. It conforms to the N-W model^46^. The complete process of the [110]_BCC_→[110]_FCC_ transformation can thus be expressed as:

1 $${\mathrm{[110]}}_{{\mathrm{BCC}}}\mathop { \to }\limits^{{\mathrm{Pitsch}}} {\mathrm{[100]}}_{{\mathrm{FCC}}}\mathop { \to }\limits^{{\mathrm{Bain}}} {\mathrm{[100]}}_{{\mathrm{BCC}}}\mathop { \to }\limits^{{\mathrm{N - W}}} {\mathrm{[110]}}_{{\mathrm{FCC}}}$$

The possibility of this transformation at room temperature was also tested in MD simulation. Supplementary Fig. 7 and Supplementary note 7 presents an MD analysis comparison of the behavior of a W single crystal during tensile deformation in the [$\bar 1$12]_BCC_ direction at 293 K and 973 K, as viewed from the [110]_BCC_ direction. No phase transformation was induced at 293 K, in clear contrast to the case at 973 K. This implies that a high temperature is essential for the strain-induced BCC→FCC phase transformation in W.

The full process from crack propagation to rupture was recorded, as presented in Supplementary Fig. 8 and Supplementary note 8. It was found that an ~30 nm wide region ahead of the crack tip was continuously transformed from the BCC to the FCC structure.

### Strain analysis of strain-induced BCC→FCC phase transformation

It is evident in Figs. 3 and 4 that a strain-induced BCC→FCC phase transformation occurred at the crack tip. The motivation for such a mechanically induced transformation was obviously the need and the ability to produce lattice dimensional change in the direction of the applied stress. This phase transformation is diffusionless, and thus, its lattice change does have the ability to provide lattice dimensional changes in response to a stress. Supplementary Fig. 9 presents a crystallographic calculation of the linear strains in the stress direction associated with the [110]_BCC_→[100]_FCC_→[100]_BCC_→[110]_FCC_ transformation processes as the Pitsch, Bain and N-W crystallographic models^46,50,52^. The results are summarized in Supplementary Table 1 together with the lattice volume changes. The transformation process provides 11.1%, 10.2%, and 28.3% elongation strains over the three steps, totaling >50% effective strain in the loading directions ([$\overline 1$12]_BCC_, [011]_FCC_, and [001]_BCC_). The BCC ↔ FCC transformation was also associated with a volume change of 5.56%.

To understand how the large lattice strains that occur during the three phase transformation stages are accommodated within the matrix, a MD simulation was carried out, and the findings are presented in Supplementary Fig. 10 and Supplementary note 9. The simulation was conducted under the same conditions as presented above. Supplementary Fig. 10a represents the initial condition before deformation. Supplementary Fig. 10b shows the distribution of the atomic strains along the [$\bar 11$2]_BCC_ direction after the [110]_BCC_→[100]_FCC_ phase transformation when the global strain was 5.1%. The atomic strain in the [100]_FCC_ region reached 11.1%. After the [100]_FCC_→[100]_BCC_ phase transformation, the atomic strain in the [100]_BCC_ region reached 22.4% (this strain is the accumulation of those of both stage 1 and stage 2) when the global strain was 5.3%, as presented in Supplementary Fig. 10c. When the global strain reached 28%, the [100]_BCC_→[110]_FCC_ phase transformation occurred, and the atomic strain in the [110]_FCC_ region was 57%, as presented in Supplementary Fig. 10d.

### Dislocation activities within the newly formed FCC phase at the crack tip

**Fig. 5:**
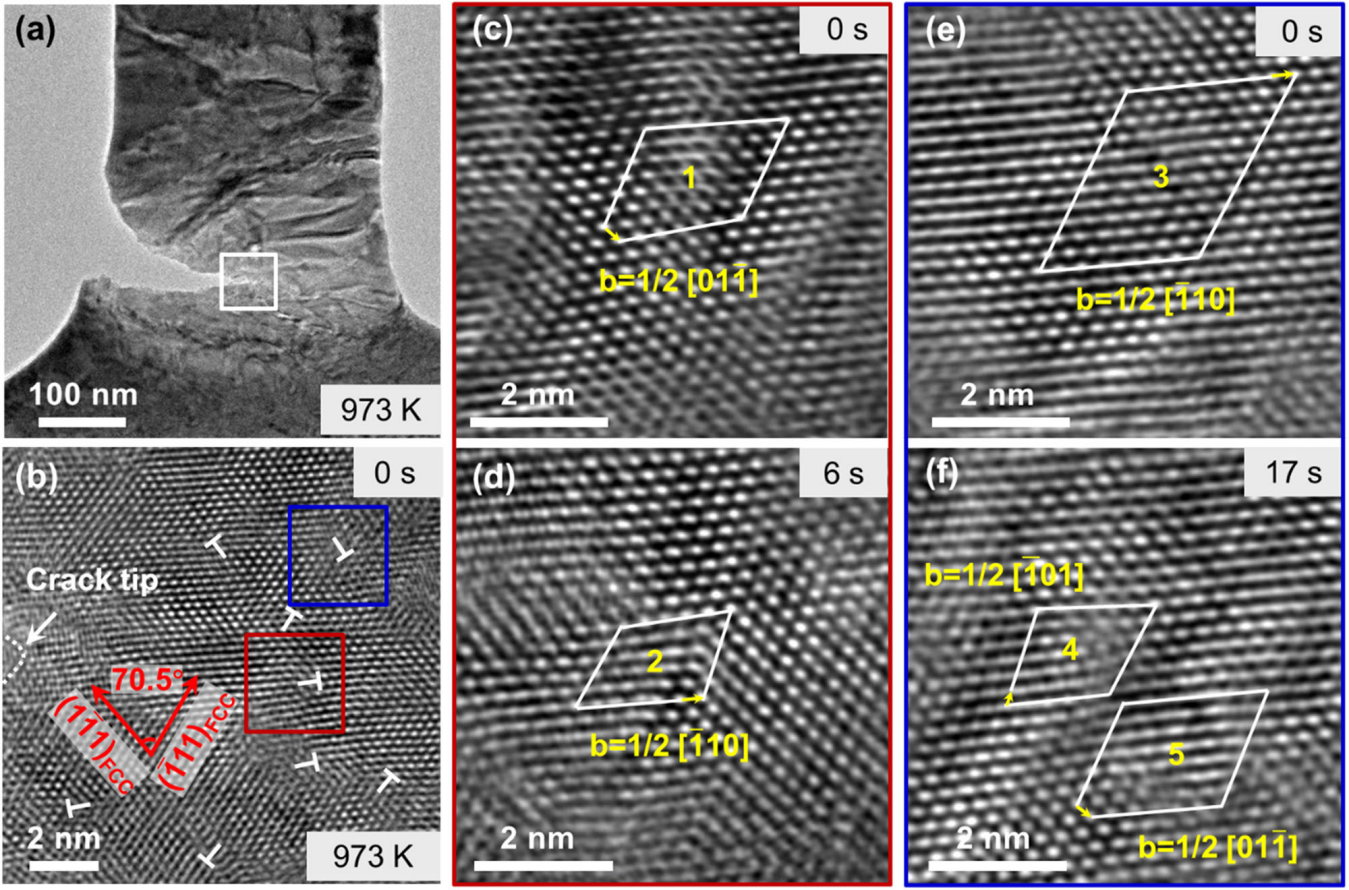
TEM analysis of dislocation activities within the FCC phase region. **a** A TEM bright-field image of a crack formed at 973 K. **b** A HRTEM image of the region within the white box in **a** in front of the crack tip. The entire region is in FCC structure in [110] normal orientation. **c** and **d** HRTEM images of the area identified by the red box in **b** at a higher magnification. The two images were taken in situ during straining. The 1/2[01$\overline 1$] dislocation seen in **c** evolved into 1/2[$\overline 1$10] dislocation after straining. **e** and **f** HRTEM images of the area identified by the blue box in **b** at a higher magnification. The two images were taken in situ during straining. The 1/2 [$\overline 1$10] dislocation in **e** decomposed into 1/2 [$\overline 1$01] and 1/2 [01$\overline 1$] dislocations after further straining.

It is also evident in Fig. 3f that the FCC structure was retained without reverting to the thermodynamically more stable BCC structure^29,31,52^ upon the relaxation of the local stresses with the propagation of the crack. To reveal the underlying mechanism, the atomic arrangement within the FCC structure was examined, as presented in Fig. 5. Figure 5a shows the location of the crack and the area of examination (within the white box) in the sample. Figure 5b shows a high-resolution image of the area in front of the crack tip. The area was in the FCC structure in the [110] zone axis, as indicated by the lattice vectors. Several dislocations can be recognized. The dislocations were all 60° full dislocations, as determined using Burger’s loops. Figure 5c shows the area identified by the red box in Fig. 5b in detail. The dislocation in this area (designated number “1”) had a Burger’s vector of **b**_**1**_ = ½[01$\bar 1$]. Figure 5d shows the same area after 6 s of straining from the state shown in Fig. 5c. The dislocation (designated number “2”) changed to a **b**_**2**_ = ½[$\bar 1$10] full dislocation.

Figure 5e shows in detail the area identified by the blue box in Fig. 5b. The dislocation in this area (designated number “3”) had a Burger’s vector of **b**_**3**_ = ½[$\bar 1$10]. Upon further straining for ~17 s from (e), the dislocation decomposed into two new 60° full dislocations marked as “4” and “5”, as shown in Fig. 5f. Their Burger’s vectors were **b**_**4**_ = ½[$\bar 10$1] and **b**_**5**_ = ½[01$\bar 1$]. This dislocation reaction can be expressed as:

2 $$1/2\left[ {\bar 1{\mathrm{10}}} \right] \to 1/2\left[ {\bar 1{\mathrm{01}}} \right] + 1/2\left[ {{\mathrm{01}}\bar 1} \right]$$

These dislocations and their movements broke up the long-range order in the FCC structure and may have served as pinning sites to the FCC structure to prevent its reversion to BCC when the stress was relaxed by the arrival of the crack tip^53^. The pinning effect may be attributed to the energy required by the dislocations for the reverse FCC→BCC transformation. In a diffusionless FCC→BCC transformation, a **b** = ½[110]_FCC_ dislocation in the FCC structure is transformed to a **b** = [001]_BCC_ dislocation in the BCC structure. According to the dislocation elastic strain energy equation:

3 $$W = \alpha G{\mathbf{b}}^2$$

where *α* is a geometrical coefficient, the energy of the **b** = ½[110]_FCC_ dislocation in the FCC structure is 8.24*αG* and that of the **b** = [001]_BCC_ dislocation in the BCC structure is 10.02*αG*, the latter being higher than the former. Thus, when there was a sufficient density of **b** = ½[110]_FCC_ dislocations in the FCC structure, it stabilized, and the FCC→BCC transformation became energetically unfavorable or thermodynamically prohibited.

In MD simulations, the FCC phase reverses back to the BCC phase in the wake of a passing crack, when the internal stresses are relaxed. Supplementary Fig. 11 and Supplementary note 10 shows an MD simulation of the BCC-FCC transformation induced by crack propagation in W at 973 K. It is evident that the volume fraction of the FCC phase increases during early deformation but some of the FCC phase has disappeared with extensive cracking (when the stress is relaxed). In addition, it also evident that dislocations are formed in both the BCC phase and the newly formed FCC phase. The dislocations in the FCC phase appear to help to stabilize and retain the thermodynamically unstable FCC phase from reversing back to the BCC phase upon unloading (stress relaxation), as seen in Supplementary Fig. 11d

### Reducing the stress concentration at the crack tip

**Fig. 6:**
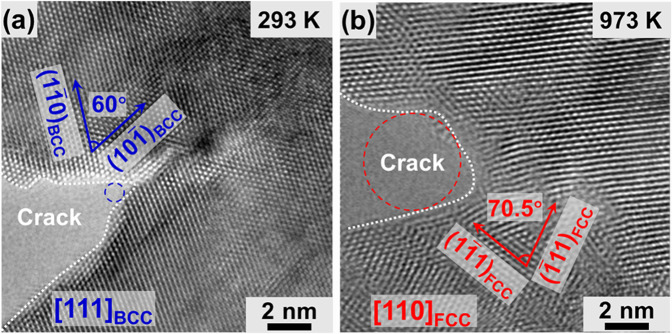
Stress distribution analysis at the crack tip. **a** HRTEM image of the crack tip in a sample deformed at the room temperature. A blue circle <1 nm in diameter fitted the tip space is used to show the sharpness of the crack. **b** HRTEM image of the crack tip in a sample deformed at 973 K. A red circle with a diameter of 3 nm fitted the tip space is used to show the sharpness of the crack.

The sharpness of a crack tip is a practical indicator of the brittleness of cracking^23,54,55^. Figure 6 shows a comparison of the crack tip morphology of two samples tested at different temperatures. Figure 6a shows a sample stretched at room temperature. The structure at the crack tip remained BCC without any phase transformation. The crack tip was at an acute angle of ~48°, and the tip space was fitted with a circle <1 nm in diameter. The structural changes in the matrix and the dislocation activities at the crack tip during the crack propagation are shown in Supplementary Fig. 12 and Supplementary note 11. The plastic deformation at the crack tip at room temperature is dominated by dislocation activities, and no BCC-FCC phase transformation is found at the crack tip during the crack propagation process. The deformation of the crack tip at room temperature is similar to that of tungsten nanowires at room temperature^5^. Both of them are dominated by 1/2 < 111 > -type mixed dislocations, which are probably half dislocation loops on (0$\overline 1$1)_BCC_ planes, as seen in Supplementary Fig. 12b–d. The crack propagation mode is mainly cleavage fracture, and the cleavage planes are (0$\overline 1$1)_BCC_ and (10$\overline 1$)_BCC_, as shown in Supplementary Fig. 12d. Figure 6b shows the sample stretched at 973 K. The structure in the area near the crack tip transformed to FCC. The crack tip profile was blunt with an open space that can easily fit a circle with a diameter of 3 nm. The bluntness of the crack tip in the sample tested at the high temperature was attributed to the occurrence of the strain-induced BCC→FCC transformation and dislocation activities within the newly formed FCC phase. A blunt tip also implies a lower stress concentration. Supplementary Fig. 13 and Supplementary note 12 presents an ANSYS finite element analysis of the stress concentration comparison between the two crack tip profiles. The maximum stress in front of the crack formed at 293 K was 10,866 MPa and that of the crack formed at 973 K was 1522 MPa when applying a stress of 500 MPa to the sample along the tensile direction.

In conclusion, the evidence presented above demonstrates that the W single-crystal fractures in a ductile manner at 973 K and the mechanisms of this fracture comprise a strain-induced BCC→FCC transformation at the crack tip and dislocation activities in the FCC structure that is formed. This is enabled by a unique technique involving HRTEM analysis during in situ tensile deformation at high temperatures. These observations provide a new understanding of the mechanisms of ductile fracture of W that adds to the common knowledge of the enhanced mobility of screw dislocations at high temperatures in BCC metals^17,25,56^. The BCC→FCC phase transformation at the crack tip and the dislocation activities within the FCC phase help to absorb deformation energy and reduce local stress concentrations, thus retarding crack propagation and causing ductile fracture. It is reasonable to also expect that this mechanism is applicable for other highly brittle BCC metals at high temperatures.

## Methods

### Sample preparation

The material used was a single-crystal tungsten ingot of 99.9 wt. % purity, supplied by Goodfellow Cambridge Limited. Samples for high-resolution transmission electron microscopy (HRTEM) examination were fabricated by means of focused ion beam (FIB) milling using an FEI Helios 600i FIB instrument. The sample was fabricated into a dog-bone shape for in situ tensile deformation within the microscope. The gauge dimensions of the samples were 1200 × 300 × 60 nm.

### Experimental methods

TEM observations were conducted on a FEI-ETEM transmission electron microscope at 300 kV. Time-resolved HRTEM images were recorded on digital video at a speed of five frames per second. The sample was heated at a rate of 20 K/min to 973 K and held for 15 min to stabilize before the in situ deformation. The in situ deformation of the sample was conducted using a self-developed device adopted to a TEM sample holder. The device technology has been transferred to Bestron (Beijing) Science and Technology Co., LTD. and marketed as INSTEMS-MT. The device contained two essential units, one for temperature control and the other for applying deformation (Fig. 1). Heating of the sample was achieved using a build-in micro-heater controlled by a microelectromechanical systems (MEMS) chip. The temperature of the sample was determined by measuring the power variation of the heating element using the MEMS chip, and the temperature control was achieved based on a power-temperature calibration curve to an accuracy of ±5 K. The unit for deformation was driven by a lead zirconate titanate (PZT) actuator under displacement control. The PZT actuator was able to provide a full displacement range of 4 µm with a step size of 0.1 nm (corresponding to a strain resolution of ~10^−4^ relative to the sample size used), a maximum load on the order of 1 N (corresponding to a maximum stress of ~ 4 GPa for the sample size used) with a 1 nN resolution. These units were built on a double-tilt sample holder that was also developed in-house. The holder can tilt the sample about the *x* and *y* axes for up to ±15°. This capability is essential to allow good atomic-scale resolution, which requires precise alignment of the electron beam to a low-index zone axis of the sample during thermomechanical testing.

### MD simulation methods

Molecular dynamics (MD) simulation was conducted using the large-scale atomic/molecular massively parallel simulator (LAMMPS) open-source code^57^ with the embedded atom potential EAM^58^. The dimensions of the sample used for simulation were 32, 10 and 10 nm along the *x* [$\overline 1$12], *y* [$\overline 1$1$\overline 1$], and *z* [110] axes, respectively. It contained approximately 200,000 atoms in total. In the simulation process, uniaxial tension was applied along the *x*-direction at a constant strain rate until failure. The simulation was conducted at 973 K. For comparison, two supercells of 30 × 10 × 10 nm in the [$\overline 1$12], [$\overline 1$1$\overline 1$], and [110] (normal) orientation containing ~200,000 atoms were used for the simulation. In the simulation process, uniaxial tensions were applied along the [$\overline 1$12]_BCC_ direction at a constant strain rate until failure. The simulations were conducted at 973 and 293 K. The atoms were colored according to their local structural environment using the polyhedral template matching (PTM) method^59^ in the OVITO software^60^.

## Supplementary information

Supplementary Information

## Data Availability

The authors declare that the main data supporting the findings of this study are available within the article and its Supplementary Information files.
