# Supplementary material for: Timely and atomic-resolved high-temperature mechanical investigation of ductile fracture and atomistic mechanisms of tungsten
Source: Nat Commun. 2021 Apr 13;12:2218. doi: 10.1038/s41467-021-22447-y (PMC8044182; doi:10.1038/s41467-021-22447-y)
Supplement: Supplementary file 1 — Supplementary Information [file 41467_2021_22447_MOESM1_ESM.pdf]

Supplementary Information

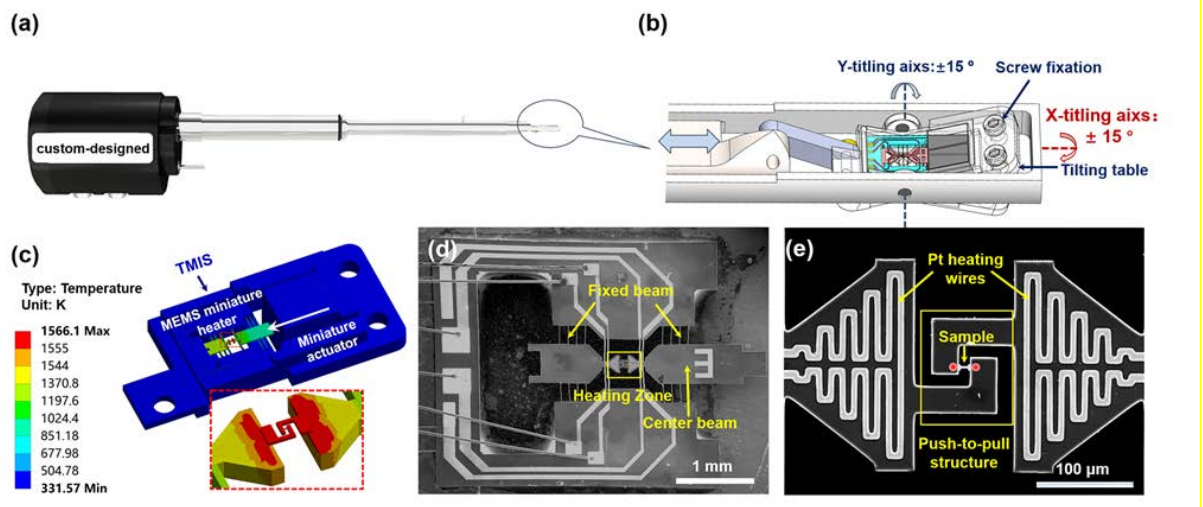

Supplementary Fig. 1: A custom designed in situ thermomechanical TEM sample holder. **(a)** The custom-designed in situ thermomechanical TEM sample holder. **(b)** Schematic diagram of the front end of the in situ thermomechanical TEM sample holder. The thermomechanical integrated stage (TMIS) is fixed on the tilting table of the sample holder. The TMIS allows the miniature actuator and the MEMS miniature heater to tilt with the sample at the same time. **(c)** ANSYS simulation of temperature distribution in the TMIS at 1556 K. The right panel is an enlarged figure of the temperature distribution in the holder and actuator zone, center-beam zone, heating zone and the sample zone, respectively. **(d)** SEM image of the MEMS miniature heater. **(e)** SEM image of heating zone and push-to-pull structure of the MEMS chip. The red dots mark the measurement positions of temperature calibration by Raman spectroscopy.

Supplementary note 1: Supplementary Fig. 1 shows the custom-designed *in situ* thermomechanical TEM sample holder used in this study[1,2]. The sample holder allows concurrent sample tilt operation and the tensile deformation of the sample at an elevated temperature, whist atomic-level high resolution TEM imaging. The MEMS heating chip and the miniature PZT actuator were built on a miniature stage to form a thermomechanical integrated stage (TMIS)[2], as shown in Supplementary Fig. 1(c). The TMIS was fixed on a double-tilt sample holder (Fig. 1(e)), also developed in-house, as shown in Supplementary Fig. 1(b). Supplementary Fig. 1(d) shows an SEM image of the MEMS heating chip. There are 12 pairs of symmetrical fixed beams in the MEMS chip to ensure a uniaxial loading of the tensile sample. The stretched sample is lift-out from a bulk sample with predetermined crystal orientation and accurately transferred to the push-to-pull unit (the two “L” shaped sample mounting arms) through an easy-lift probe that can realize nanometer-level displacement control in FIB, as shown in Fig. 1(a) and Supplementary Fig. 1(e). The 5-dimensional movable mobile stage and easy-lift probe in FIB ensure that the position accuracy of the sample is 10 nm and the angle between the desired crystallographic stretching direction of the sample and the force loading direction is less than 5°. After the sample is loaded, sample milling and dog-bone shaping are completed through the sample preparation process shown in Fig. 1(a). The displacement was applied using a miniature PZT actuator, as seen in Supplementary Fig. 1(c). The displacement was controlled (but not measured) by controlling the applied voltage to the actuator based on a calibration. The accuracy of displacement control by the PZT actuator is 0.1 nm, for a length of 1000 nm of the sample, the corresponding strain accuracy can be about

0.01%.

The alignment of the sample with the loading direction was ensured in two aspects: The first aspect is the alignment of the loading apparatus including the PZT actuator and the MEMS' sample loading carrier, to ensure that the loading direction is along the center beam of the MEMS chip. The loading direction was controlled by aligning the center beam of the MEMS chip to the actuation loading direction of the miniature PZT actuator during assembly of the two. Under optical microscope, 12 sets of fixed beams stretching from the center beam to the MEMS frame corrects any misalignment between the actuation of the PZT and the MEMS carrier. The second is to load the sample on the MEMS carrier. This is processed in a dual beam SEM. By visually operating the 5-dimensional movable mobile stage and the easy-lift probe, the sample cut, transfer, loading and alignment are carried out sequentially. The sample thinning direction is well controlled to be parallel to the center beam in Z direction during sample ion milling which ensures that the designed sample crystallographic orientation be aligned well with the TEM lamina sample. The tensile axis of the dog-bone shaped tensile sample is thus well aligned to be parallel to the center beam of the MEMS.

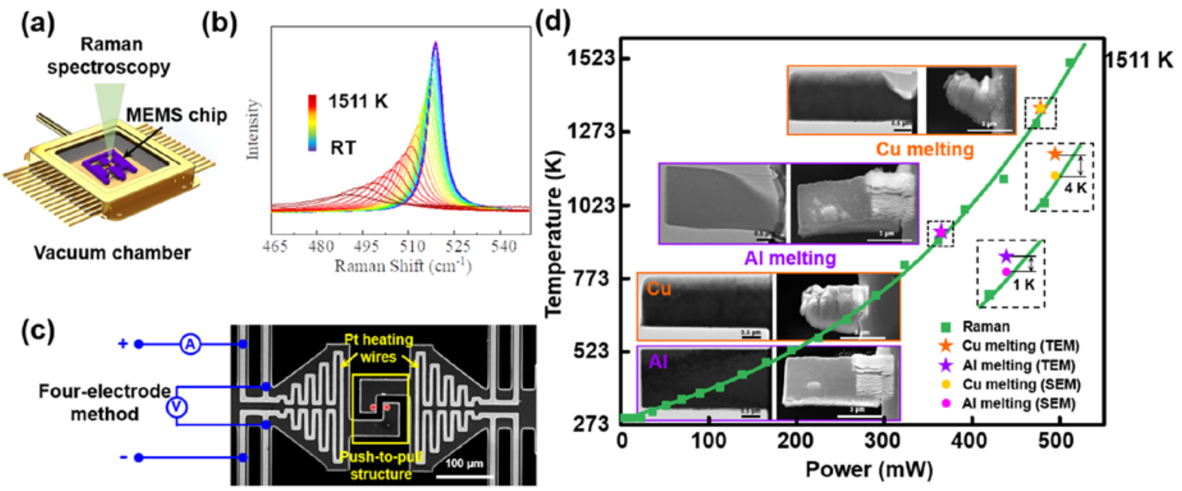

Supplementary Fig. 2: Temperature measurement and calibration of MEMS chip. **(a)** Raman spectroscopy for temperature calibration and the vacuum chamber. **(b)** Raman stokes peaks of Si at different temperatures. **(c)** A four-electrode temperature measuring device integrated in the heating zone of the MEMS chip. **(d)** Temperature-power function established empirically by Raman spectroscopy and the calibration by the melting of Al and Cu samples inside the TEM and SEM, respectively.

Supplementary note 2: Supplementary Fig. 2 shows temperature measurement and calibration of MEMS chip. The calibration of temperature was measured by means of Raman Spectroscopy in a vacuum chamber (better than 10-4 Pa), as shown in Supplementary Fig. 2(a). The temperature control and measurement are realized by the four-electrode method, as shown in Supplementary Fig. 2(c). The measurement was conducted at the tips of the single-crystal Si mounting arms for the sample, as indicated by the red dots in Supplementary Fig. 2(c). Supplementary Fig. 2(b) shows the Raman stokes peak of Si at different temperatures. The temperature-power curve was obtained by Raman spectroscopy and observing the melting of

Al and Cu samples inside the TEM and SEM, as shown in Supplementary Fig. 2(d).

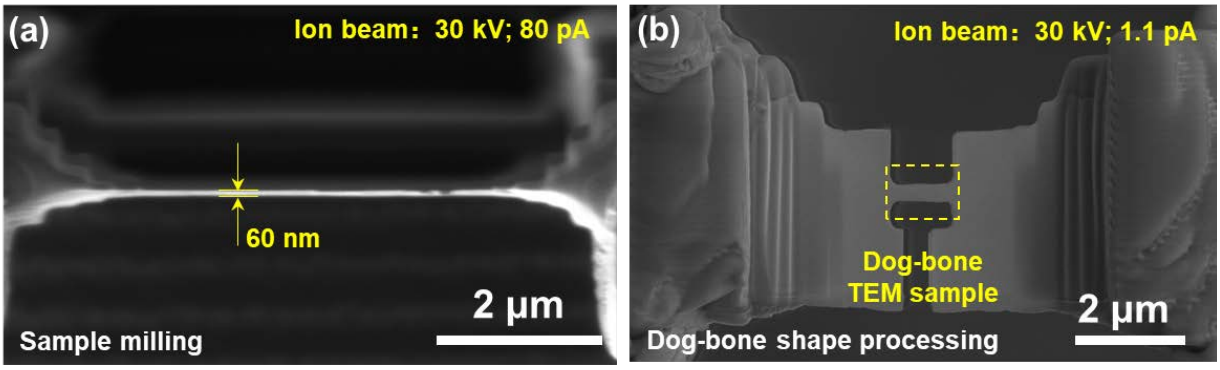

Supplementary Fig. 3: The process of sample preparation by FIB. **(a)** SEM image of the side view of the sample under milling from both sides. **(b)** SEM image of the top view of sample being cut into dog-bone shape.

Supplementary note 3: Supplementary Fig. 3 shows the process of sample preparation by means of FIB milling technique. In the process, a slice W of 10×5×2 μm^3 in dimension and $[110]_{BCC}$ normal oriented and $[\bar{1}12]_{BCC}$ in length direction was first extracted from a single crystal W bulk and then loaded on the MEMS heating chip by an easy-lift needle. The length direction of the slice was aligned to the stretching direction of the push-to-pull device. The slice sample was then welded onto the Si push-to-pull arms by ion beam-induced deposition (EBID) using a Pt nanocomposite material (nanometer-sized Pt metal crystals in an *a*-C matrix). Supplementary Fig. 3(a) shows an SEM image of the sample under milling from both sides to reduce its thickness. The thickness of the sample was reduced from 2 μm to 60 nm using a 30 kV, 80 pA ion beam. The thinned sample was then milled into the dog-bone shape using a 30 kV, 1.1 pA ion beam, as shown in Supplementary Fig. 3(b). Finally, a 5 kV, 7 pA ion beam was used to clean the surfaces of the shaped sample at a glancing angle of ±4° to remove Ga contaminants and damaged layers.

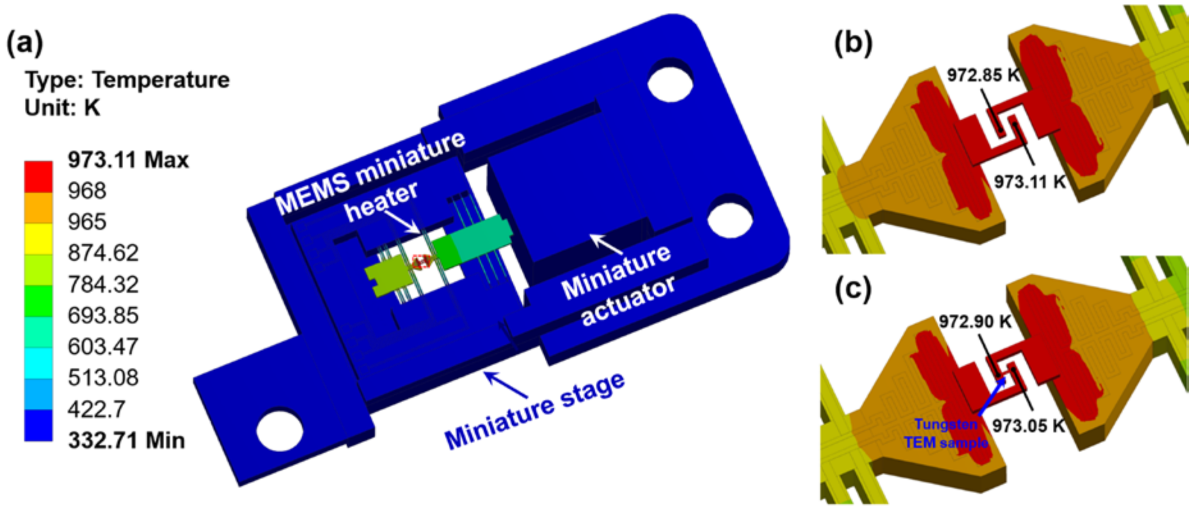

Supplementary Fig. 4: ANSYS simulation of the influence of the TEM sample on temperature distribution. (a) The ANSYS temperature distribution simulation of the thermomechanical integrated stage (TMIS) at 973 K. (b) The temperature at both ends of the push-to-pull structure when there is no sample at 973 K. (c) The temperature at both ends of the push-to-pull structure when the W sample is loaded at 973 K.

Supplementary note 4: Supplementary Fig. 4 shows an ANSYS finite element simulation of the temperature field around the sample, the MEMS heater and the surrounding structure. The simulation indicates that the addition of an *in situ* sample caused a temperature change of 0.06 K at the spots of temperature calibration and can be neglected.

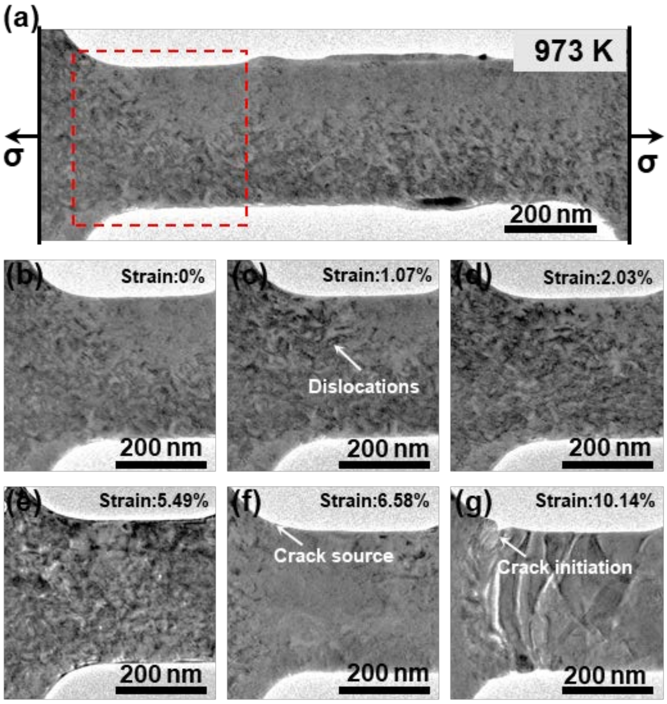

Supplementary Fig. 5: TEM images of the left end of the sample at different global strain levels before the initiation of the first crack at 973 K. **(a)** Full view of the specimen at 973 K before deformation. **(b)-(g)** TEM images of the left end of the specimen at different global strain levels up to 10.14%.

Supplementary note 5: Supplementary Fig. 5 shows the dislocation activities in the specimen during deformation at 973 K prior to the initiation of the first crack. During deformation up to 5.48%, the dislocation density continued to increase, as seen in Supplementary Fig. 5(b)-(e). At 6.58% of strain, the first crack initiated, and the local stresses are immediately relaxed and dislocation density within the vicinity of the crack reduced, as evident in Supplementary Fig. 5(f). This demonstrates that local dislocation activities are the main mechanism of deformation before the formation of cracks.

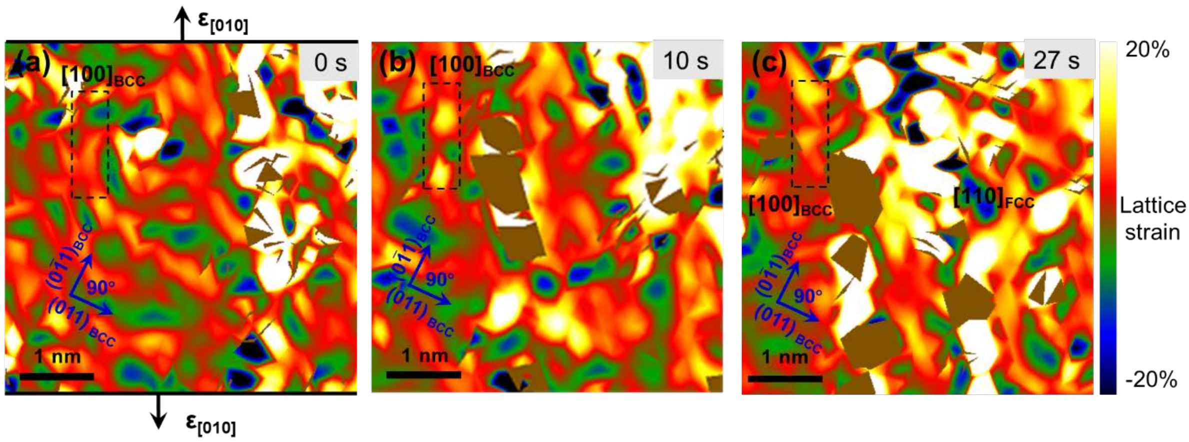

Supplementary Fig. 6: Lattice $[010]_{BCC}$ *d*-spacing strain ($\varepsilon_{[010]}$) evolution in front of a crack tip during the process of tensile straining. The lattice tensile strain maps **(a)-(c)** correspond to the HRTEM images shown in Fig. 3(c)-(e), respectively. The map images are in $[100]_{BCC}$ zone axis. The tensile direction is $[010]_{BCC}$ and the elongation rate applied was 0.1 nm/s.

Supplementary note 6: The $[010]_{BCC}$ *d*-spacing strain maps shown in Supplementary Fig. 6 are determined using the Peak Pairs Analysis method. The area in the dashed box is at the interface between $[100]_{BCC}$ and $[110]_{FCC}$ regions. Strain map **(a)** represents the situation of the crack tip under stress during crack initiation, record this moment as 0s. The angle between the two basic lattice vectors are 90° and the average $[010]_{BCC}$ *d*-spacing strain is 1.0%. Strain map **(b)** corresponds to the condition after 10 seconds of loading. The angle between the two basic lattice vectors is 81° and the average $[010]_{BCC}$ *d*-spacing lattice strain reaches 1.5%. Strain map **(c)** represents the moment 27 seconds after the continuous loading. The angle between the two basic lattice vectors has changed to 70.5° and the BCC → FCC phase transformation has occurred. The average $[010]_{BCC}$ *d*-spacing strain has reached 3.8%.

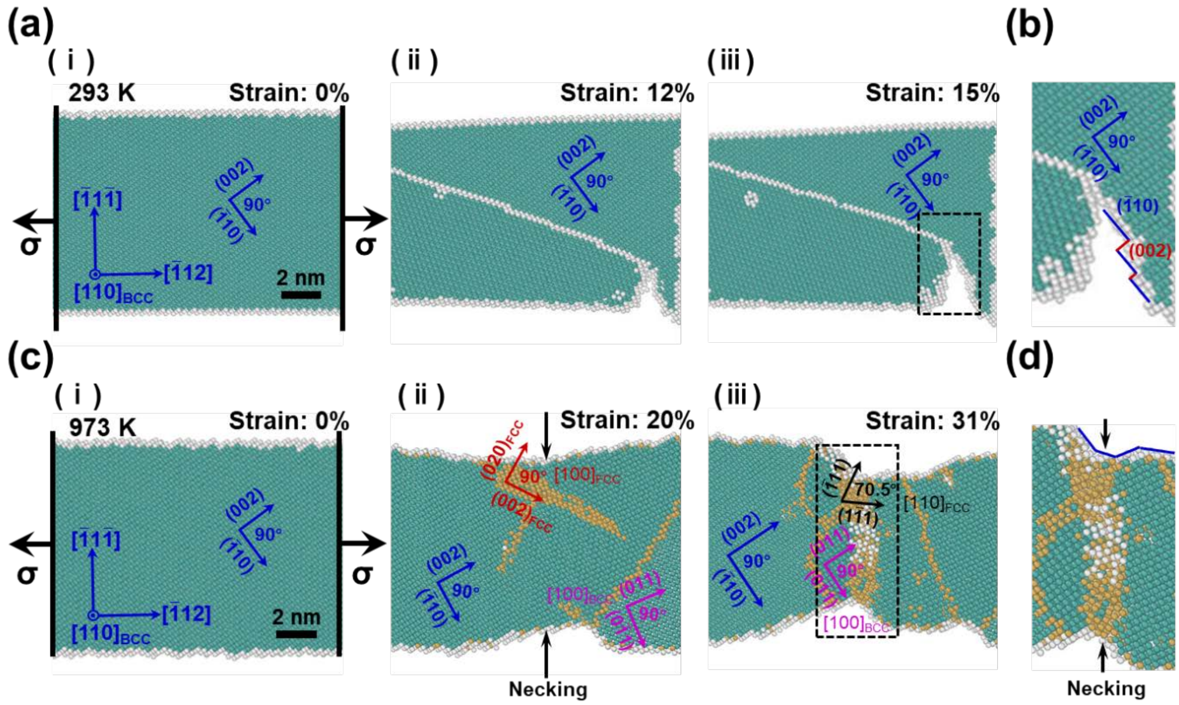

Supplementary Fig. 7: Molecular dynamics simulation of the crack growth behavior and structural changes of the BCC lattice at 293 K and 973 K during tension. The viewing direction

is [110] and the stretching direction is [$\bar{1}$12]. **(a)** The crack propagation process at 293 K. **(b)** An enlarged view of the crack showing the ($\bar{1}$10) and (002) cleavage planes. **(c)** The crack propagation process at 973 K. Different crystal phases are presented in different colors. **(d)** An enlarged view of the heavily necked region.

Supplementary note 7: When stretched at 293 K, no phase transformation was observed in front of the crack tip during crack propagation. The sample fractured in brittle manner by cleavage along ($\bar{1}$10) and (002) planes. When deformed at 973 K, BCC → FCC transformation occurred at the crack tip, as indicated by the orange colored regions. Heavy lateral necking occurred as a result of the significant elongation in the stress direction associated with transformation instead of cracking, demonstrating the ductile manner.

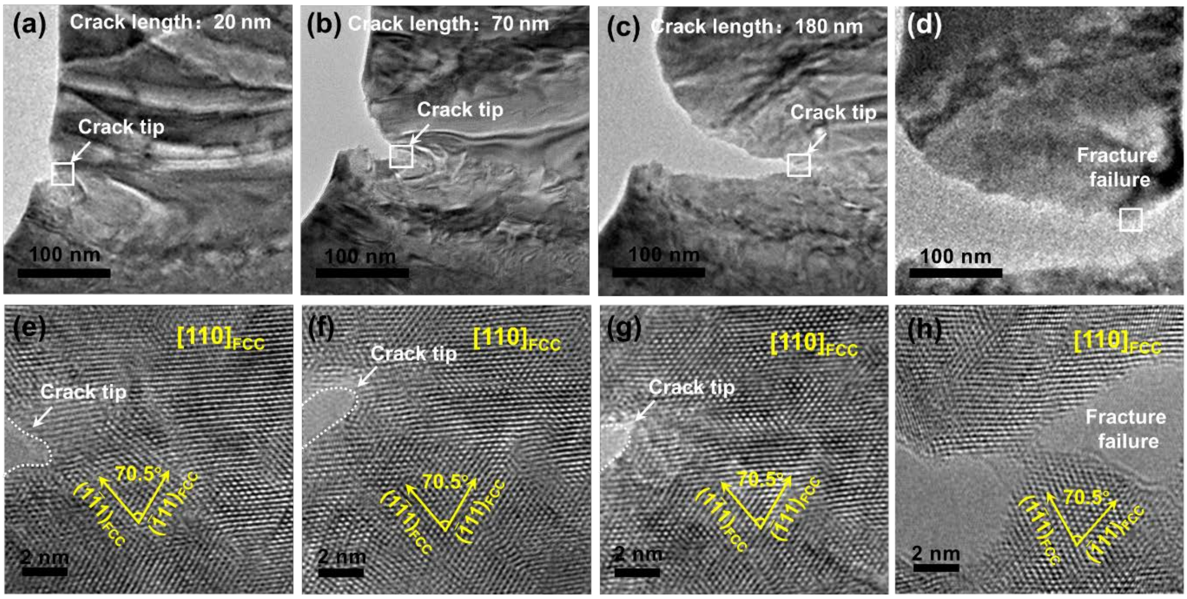

Supplementary Fig. 8: The full process of a crack propagation in single crystal W at 973 K. The HRTEM images shown in **(e)** ~ **(f)** corresponds to the areas identified by the small white boxes seen in **(a)** ~ **(d)**, respectively.

Supplementary note 8: The *in situ* observation of the propagation of the crack tip revealed that a ~ 30 nm wide region ahead of the crack tip always remained in the $[110]_{FCC}$ structure during the crack propagation, as evidenced by the lattice vector angle of 70.5°.

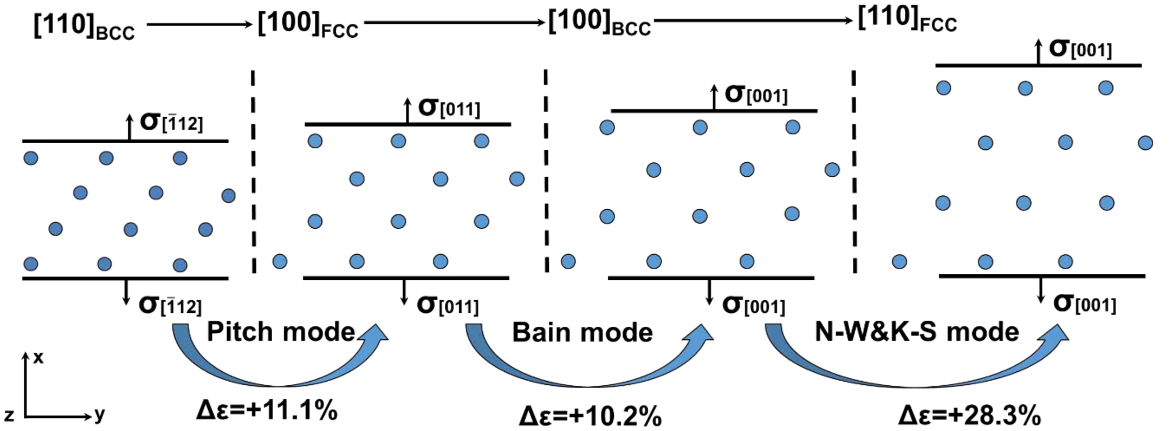

Supplementary Fig. 9: Schematic of the lattice elongation in the tension direction during the strain-induced $[110]_{BCC}$ → $[100]_{FCC}$ → $[100]_{BCC}$ → $[110]_{FCC}$ phase transformation process.

Supplementary Table 1: Lattice strains in three directions and volume changes of the $[110]_{BCC}$ → $[100]_{FCC}$ → $[100]_{BCC}$ → $[110]_{FCC}$ phase transformation process.

	d-spacing in x direction (Å)	Strain increment ($\Delta\varepsilon_x$)	*d*-spacing in y direction (Å)	Strain increment ($\Delta\varepsilon_y$)	*d*-spacing in z direction (Å)	Strain increment ($\Delta\varepsilon_z$)	Volume change (ΔV)
Z=$[110]_{BCC}$	1.292		2.741		2.238		
Z=$[100]_{FCC}$	1.435	+11.1%	2.871	+4.7%	2.030	-9.3%	+5.6%
Z=$[100]_{BCC}$	1.582	+10.2%	3.165	+10.2%	1.582	-22.1%	-5.3%
Z=$[110]_{FCC}$	2.030	+28.3%	2.874	-9.2%	1.435	-9.2%	+5.6%
Z=$[110]_{BCC}$ →Z=$[110]_{FCC}$		+57.1%		+4.9%		-35.9%	+5.6%

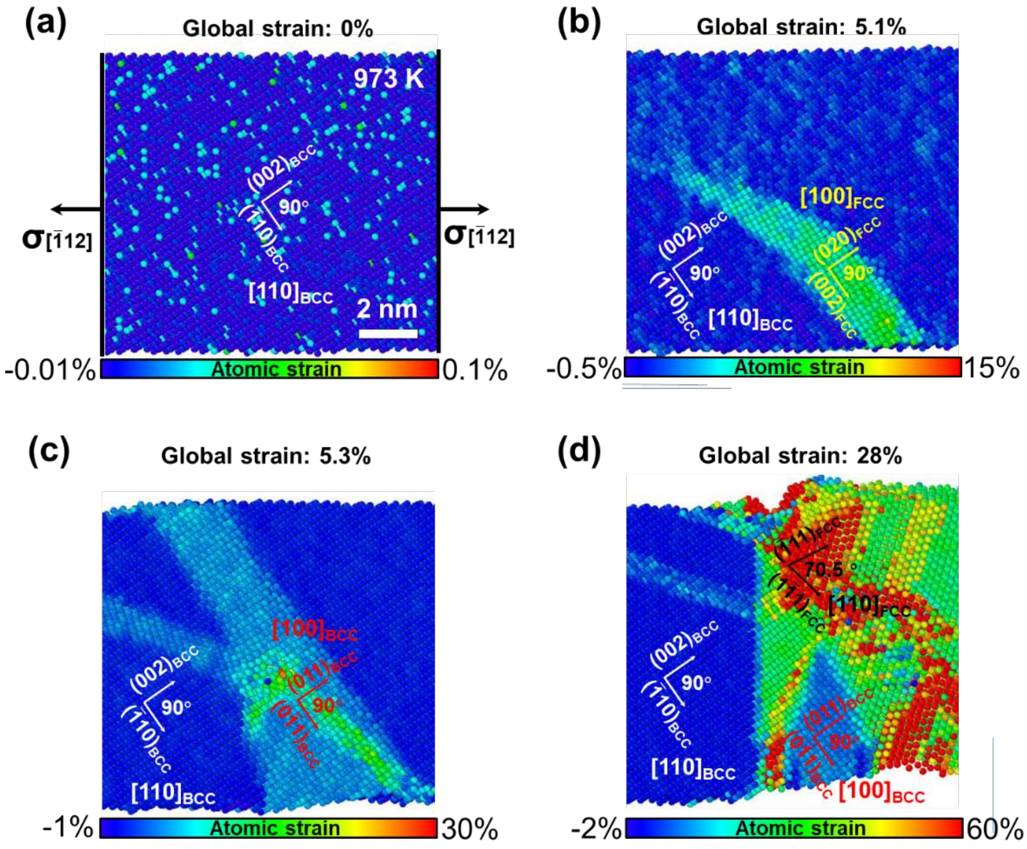

Supplementary Fig. 10: Atomic strain analysis after each phase transformation at 973 K during stretching by molecular dynamics simulation. The viewing direction is $[110]_{BCC}$. The stretching direction is $[\bar{1}12]_{BCC}$ and the strains presented are in the stretching direction. **(a)** Atomic strain mapping at 0% global strain. **(b)** Atomic strain mapping after $[110]_{BCC}$ → $[100]_{FCC}$ phase transformation, when the global strain is 5.1%. **(c)** Atomic strain mapping after $[100]_{FCC}$ → $[100]_{BCC}$ phase transformation, when the global strain is 5.3%. **(d)** Atomic strain mapping after $[100]_{BCC}$ → $[110]_{FCC}$ phase transformation, when the global strain is 28%.

Supplementary note 9: When stretched at 973 K, the three phase transformations provide net atomic strain increments of +11.1%, +10.2%, and +28.3% in the stretching direction x. Concurrently, the lattice also experiences atomic strains of +4.7%, +10.2%, and -9.2% in the y direction, and -9.3%, -22.1%, and -9.2% in the z direction over the three phase transformations, respectively (Supplementary Table 1). Such large atomic strains are found to be accommodated by the elastic distortion of both phases across the boarders. For example, for the first stage $[110]_{BCC} \rightarrow [100]_{FCC}$ transformation, which has a transformation strain of 11.1% in the stretching direction of $[\bar{1}12]_{BCC}$/ $[011]_{FCC}$, the maximum strain in the BCC phase is 3.4% and the minimum stain in the FCC phase is 4.5%, as a result of the lattice elastic distortion.

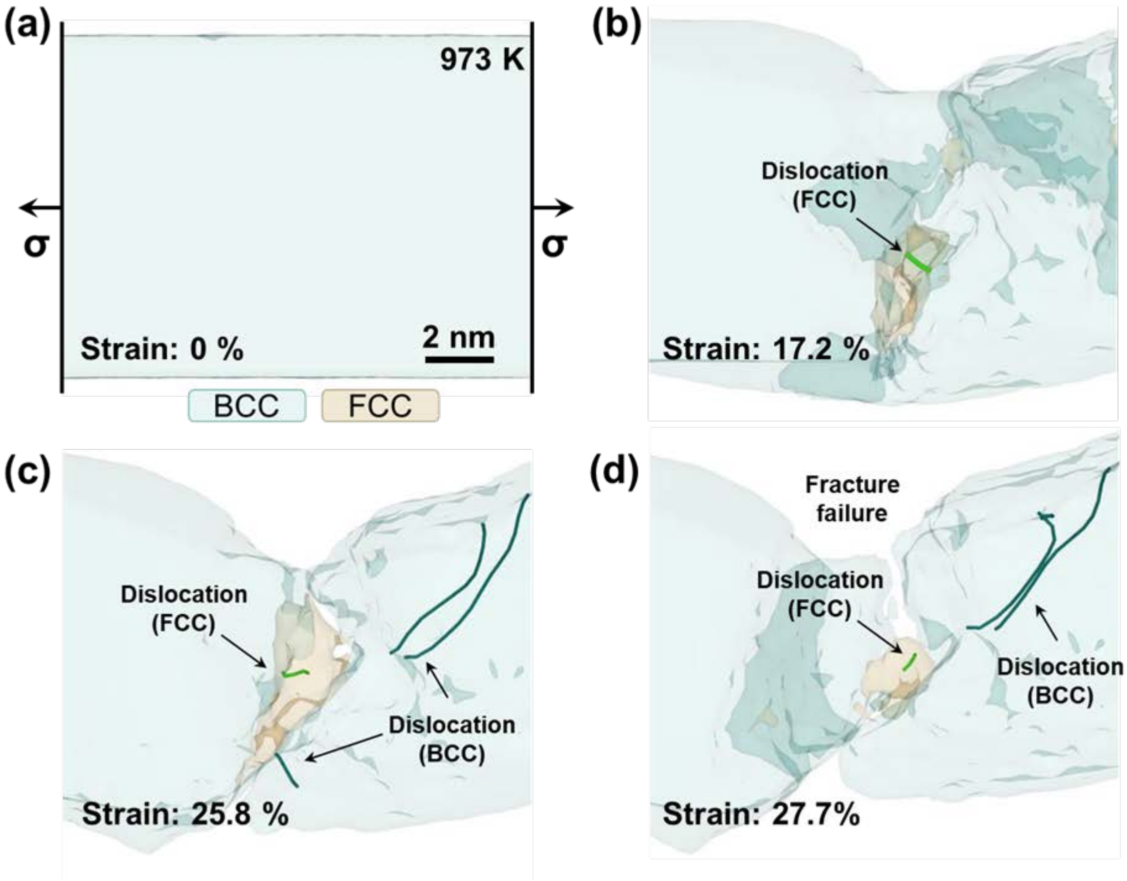

Supplementary Fig. 11: Molecular dynamics simulation of W single crystal upon stretching along $[\bar{1}12]$ at 973 K.

Supplementary note 10: Supplementary Fig. 11 shows an MD simulation of the BCC-FCC transformation induced by crack propagation in W at 973 K. It is evident that the volume fraction of the FCC phase increases during early deformation but some of the FCC phase has disappeared with extensive cracking (when the stress is relaxed). In addition, it also evident that dislocations are formed in both the BCC phase and the newly formed FCC phase. The dislocations in the FCC phase appear to help to stabilize and retain the thermodynamically unstable FCC phase from reversing back to the BCC phase upon unloading (stress relaxation), as seen in Supplementary Fig. 11(d).

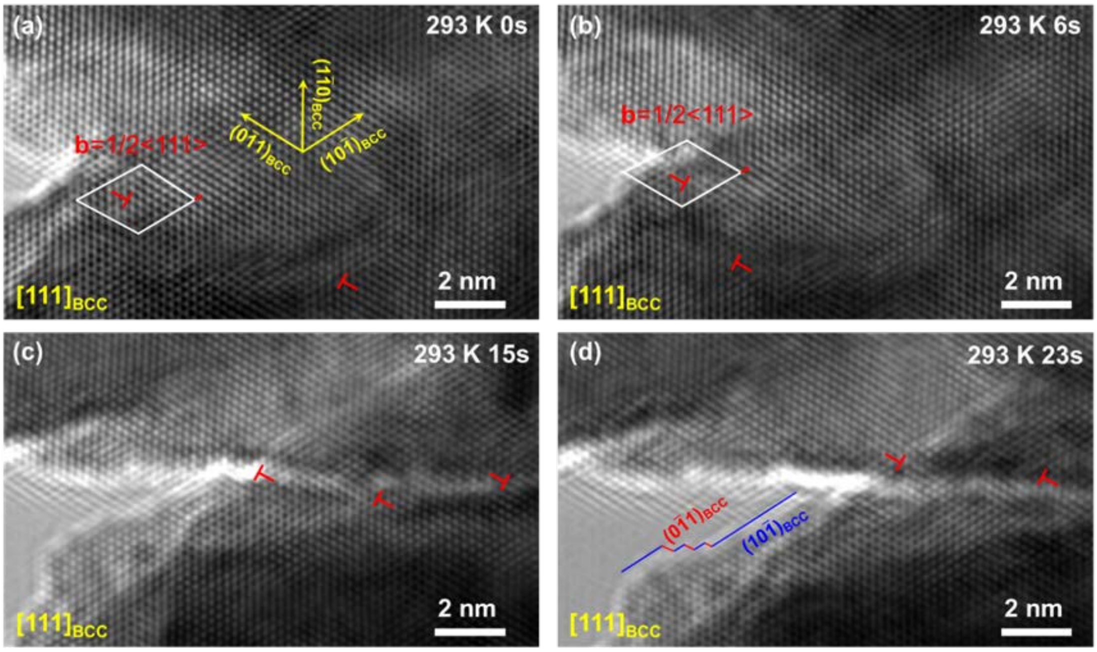

Supplementary Fig. 12: TEM analysis of dislocation activities in front of a crack tip during crack propagation in single-crystal W at room temperature. **(a)** A HRTEM image of the region in front of the crack tip. The region is in BCC structure in [111] normal orientation. **(b)-(d)** Time-resolved HRTEM images showing the structure during crack propagation and the dislocation activities at the crack tip.

Supplementary note 11: Supplementary Fig. 12 shows time-resolved HRTEM images of the crack tip during crack propagation at room temperature. Comparing the atomic structure of the crack tip at room temperature and 973 K, it can be found that the plastic deformation mechanism of the crack tip at room temperature and that at 973 K are different. The plastic deformation at the crack tip at room temperature is dominated by dislocation activities, and no BCC-FCC phase transformation is found at the crack tip during the crack propagation process. The deformation of the crack tip at room temperature is similar to that of tungsten nanowires at room temperature[3]. Both of them are dominated by 1/2<111>-type mixed dislocations, which are probably half dislocation loops on $(0\bar{1}1)_{BCC}$ planes, as shown in Supplementary Fig. 12(b)-(d). The crack propagation mode is mainly cleavage fracture, and the cleavage planes are $(0\bar{1}1)_{BCC}$ crystal plane and $(10\bar{1})_{BCC}$ crystal plane, as shown in Supplementary Fig. 12(d).

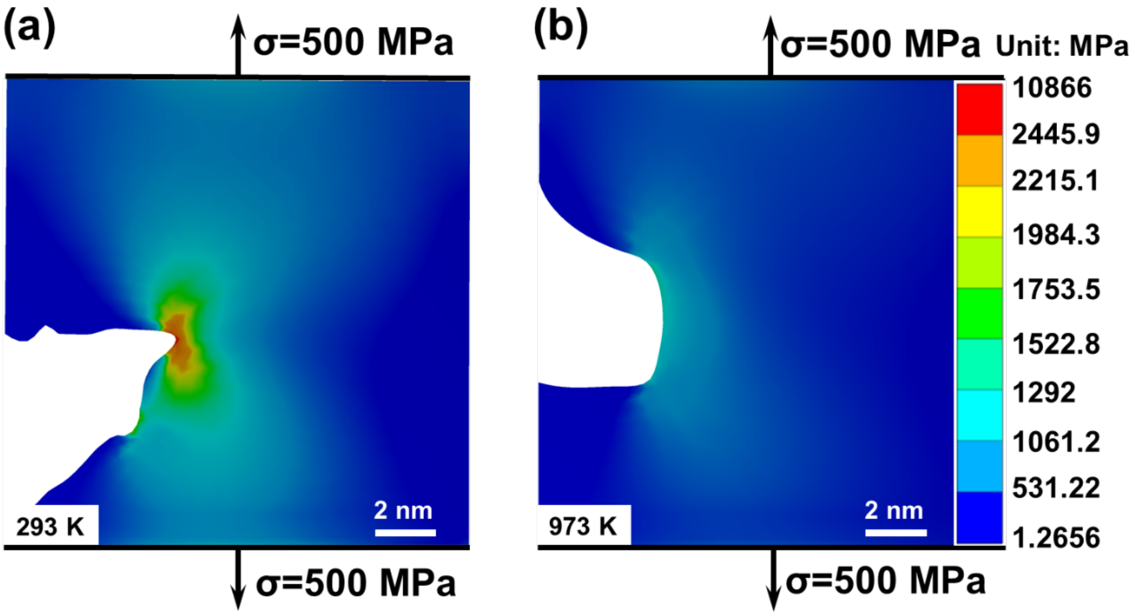

Supplementary Fig. 13: ANSYS simulation of the stress distributions around the two profiles of crack tips formed in single crystal W at the room temperature and at 973 K.

Supplementary note 12: The maximum stress for the crack formed at 293 K is 10,866 MPa and the maximum stress for the crack formed at 973 K is 1522 MPa based on the Young's modulus of 395 GPa at 293 K and 380 GPa at 973 K of W[4].

Supplementary References

1. Han, X. et al. Double-tilt sample holder for transmission electron microscope. U. S. Patent Application US10103000B2 (2018).
2. Han, X. et al. Double-tilt in-situ mechanical sample holder for TEM based on piezoelectric ceramic drive. U. S. Patent Application US10103001B2 (2018).
3. Wang, J. et al. In situ atomic-scale observation of twinning-dominated deformation in nanoscale body-centred cubic tungsten. *Nat. Mater.* **14,** 594-600 (2015).
4. Škoro, G. P. et al. Dynamic Young's moduli of tungsten and tantalum at high temperature and stress. *J Nucl Mater* **409**, 40-46 (2011).
